# Preparation and Characterization of Theasinensin A-Enriched Instant Black Tea

**DOI:** 10.3390/foods15101638

**Published:** 2026-05-08

**Authors:** Shuixin Ye, Weiwei Wang, Feilong Yang, Mengxue Zhang, Haihua Zhang, Heyuan Jiang

**Affiliations:** 1 Institute of Urban Agriculture, Chinese Academy of Agricultural Sciences, Chengdu 610213, China; 15305789331@163.com (S.Y.); yangfeilong@caas.cn (F.Y.); 2 College of Food and Health, Zhejiang A & F University, Hangzhou 311300, China; hhzhang@zafu.edu.cn; 3 Tea Research Institute, Chinese Academy of Agricultural Sciences, Hangzhou 310008, China; wangwei11211@163.com (W.W.); mengxue.zhang@h-shgroup.com (M.Z.); 4 Graduate School, Chinese Academy of Agricultural Sciences, Beijing 100081, China

**Keywords:** instant black tea, theasinensin A, response surface methodology, process optimization

## Abstract

Instant black tea is widely used in both the food and pharmaceutical industries. However, traditional water-mediated preparation leads to the loss of theasinensin A (TSA), a characteristic compound of black tea. This study aimed to optimize the preparation of instant black tea using TSA content as the primary quality indicator. Through response surface methodology, the ideal extraction parameters were identified as 49% ethanol (*v*/*v*), extraction at 70 °C for 41 min, and a material-to-liquid ratio of 1:60 g/mL. Under these optimized conditions, the TSA content reached a maximum of 456.05 ± 4.45 mg per 100 g of instant tea powder, with the predictive model showing high accuracy (only 0.3% deviation from experimental values). The resulting instant black tea presented as a dark yellow powder, yielding a dark brownish-red infusion with a characteristically strong, thick, and astringent flavor profile. Compositional analyses indicated that the proposed preparation produced an instant tea with a high total polyphenol content, particularly rich in TSA. These findings provide a theoretical foundation for developing functionally enriched instant tea, offering promising potential for nutraceutical and specialized food applications.

## 1. Introduction

Black tea accounts for approximately 78% of global tea production, a prominence attributed to its appealing qualities, such as its sweet aroma and robust flavor, as well as its recognized health benefits [1,2]. Studies indicate that black tea exhibits various physiological functions, including alleviation of colitis and liver damage [3], reduction of body fat and support for weight management [4], prevention of atherosclerosis [5], mitigation of hypertension [6], and inhibition of hepatocellular carcinoma [7]. These benefits are largely derived from bioactive constituents such as polyphenols and amino acids. Among these, theasinensins (TSs) represent an important class of dimeric compounds in black tea [8].

Among TSs, theasinensin A (TSA), theasinensin B, and theasinensin C are the dominant forms, with TSA alone accounting for over 90% of their total content [9]. TSA features stronger interflavan bonds and a more compact three-dimensional conformation, which causes its relatively low absorption rate under oral administration compared with its monomeric precursor, epigallocatechin gallate (EGCG) [8,10]. However, TSA may still exert biological functions by interacting with the gut microbiota, potentially modulating microbial composition and enhancing the production of bioactive metabolites [11]. Research has demonstrated that TSA possesses significant physiological activities, including anti-inflammatory and antidiabetic effects, as well as the ability to improve hyperlipidemia and combat obesity [12,13]. In some respects, TSA demonstrates bioactivity comparable or even superior to some catechins and theaflavins [10,14], making it a subject of growing interest in recent tea chemistry research.

Instant black tea, a solid extract obtained from black tea leaves, offers notable convenience due to its quick solubility [15,16]. Unlike traditional teas, this product dissolves directly in hot or cold water without steeping or filtration, and is therefore classified as “instant tea” (or “instant black tea”) in both academic literature and the food industry [16]. Given that TSA is an important and characteristic bioactive compound in black tea, enriching it in instant tea powder is highly desirable. However, TSA-enriched instant tea is currently lacking in the market, as conventional preparation methods typically rely on water extraction, which leads to substantial loss of TSA [17,18]. To address this limitation, alternative preparation processes are needed.

Polyphenol extraction often relies on solvents such as aqueous methanol, ethanol, ethyl acetate, and chloroform [18]. For instance, 80% aqueous acetone was reported to successfully isolate 7.6 g of TSA from fresh tea leaves, giving a yield of 0.049% [19]. Compared with aqueous acetone, aqueous ethanol offers advantages in being greener, less toxic, and food-grade, and its polarity can be adjusted to achieve comparable TSA extraction efficiency [20]. However, no study has systematically optimized aqueous ethanol extraction parameters specifically for TSA enrichment in instant black tea.

In this study, an aqueous ethanol extraction method was applied to produce instant black tea with enhanced retention of TSA. Using response surface methodology, extraction parameters were optimized. The resulting product was subsequently analyzed for its chemical composition and sensory attributes. This work establishes a foundation for developing instant black tea powders with elevated levels of TSA. The resulting preparation method for TSA-enriched instant tea was intended for nutraceutical and specialized functional food applications.

## 2. Materials and Methods

### 2.1. Materials

Jun Mei black tea was provided by Zhengshantang Co., Ltd. (Wuyi, China). The following authentic standards were used for chromatographic analysis: caffeine (CAF, ≥98%), gallic acid (GA, ≥98%), and a series of catechins including gallocatechin (GC, ≥98%), epigallocatechin (EGC, ≥98%), catechin (C, ≥98%), epicatechin (EC, ≥98%), epigallocatechin gallate (EGCG, ≥98%), gallocatechin gallate (GCG, ≥98%), epicatechin gallate (ECG, ≥98%), and catechin gallate (CG, ≥98%) were purchased from Sigma-Aldrich (St. Louis, MO, USA). Theaflavin (TF, ≥98%), theaflavin-3-gallate (TF-3-G, ≥98%), theaflavin-3′-gallate (TF-3′-G, ≥98%), and theaflavin-3,3′-digallate (TFDG, ≥98%) were obtained from Wako Pure Chemical Industries, Ltd. (Osaka, Japan). Theasinensin A (TSA, ≥98%) was kindly supplied by Nagasaki University (Nagasaki, Japan).

Common chemical reagents, including anhydrous ethanol, methanol, Folin–Ciocalteu reagent, sodium carbonate, and phosphoric acid (all of analytical grade), were sourced from McLean Biochemistry Technology Co., Ltd. (Shanghai, China). Acetonitrile (HPLC grade) was purchased from Merck (Darmstadt, Germany). Purified water was obtained from Wahaha Group Co., Ltd. (Hangzhou, China).

### 2.2. Preparation of Instant Black Tea

Instant black tea was prepared using an aqueous ethanol extraction method. Briefly, 2 g of Jun Mei black tea powder was weighed. Subsequently, 120 mL of 49% ethanol (*v*/*v*) was added, and the mixture was shaken thoroughly. The flask was then incubated in a water bath maintained at 70 °C for 41 min. Following extraction, the mixture was centrifuged and filtered. The filtrate was concentrated to 25–30% of its original volume using a rotary evaporator under reduced pressure. The concentrate was finally subjected to freeze-drying to obtain the instant black tea powder.

For comparative purposes, a control sample was prepared using the traditional hot-water extraction method [21]. In this procedure, ultrapure water served as the solvent. The extraction was conducted at 70 °C for 40 min with a material-to-liquid ratio of 1:25 (g/mL). The subsequent steps of concentration and freeze-drying were identical to those described above for the ethanol extraction method.

### 2.3. Optimization of Preparation Parameters

The effects of four key preparation parameters on TSA yield were investigated through single-factor experiments. The tested ranges were as follows: ethanol concentration (0, 20, 40, 60, and 80%, *v*/*v*), material-to-liquid ratio (1:20, 1:30, 1:40, 1:50, and 1:60 g/mL), extraction temperature (40, 50, 60, 70, and 80 °C), and extraction time (10, 20, 30, 40, and 50 min).

In a typical procedure, a measured quantity of Jun Mei black tea powder was accurately weighed into a conical flask. An ethanol aqueous solution at the designated concentration was added according to the specified solid-to-liquid ratio, and the mixture was shaken thoroughly. The flask was then placed in a thermostatic water bath and extracted for the set duration. After extraction, the mixture was centrifuged at 4000 rpm for 10 min. The supernatant was collected, made up to a fixed volume, and stored for subsequent analysis.

### 2.4. Determination of Total Polyphenols

The total polyphenol content in the tea extracts was determined using the Folin–Ciocalteu colorimetric method. In brief, the extract was reacted with Folin–Ciocalteu reagent under alkaline conditions. The absorbance of the resulting blue complex was measured at 765 nm. Quantification was performed using a gallic acid standard curve, and the results are expressed as milligrams of gallic acid equivalents per gram of dry weight (mg GAE/g).

### 2.5. Determination of Catechin, Theaflavin, and TSA Content

The quantification of individual catechins, theaflavins, and TSA was performed by high-performance liquid chromatography (HPLC), following a previously reported method [22,23] with minor modifications. Calibration curves were established using a series of prepared standard solutions. Prior to injection, all sample solutions were filtered through a 0.45 μm membrane filter. Chromatographic separation was achieved using a 5C18-AR-II column (250 mm × 4.6 mm, 5 μm) maintained at 35 °C. The mobile phase consisted of (A) 50 mmol/L phosphoric acid and (B) acetonitrile, delivered at a flow rate of 0.8 mL/min. The injection volume was 10 μL, and detection was performed at 278 nm. The gradient elution program was set as follows: phase A was decreased linearly from 96% to 70% over 0–39 min, then from 70% to 25% over 39–54 min, and finally increased from 25% back to 96% within 54–55 min for column re-equilibration.

### 2.6. Response Surface Methodology

The preparation process for TSA-enriched instant black tea was optimized using response surface methodology (RSM) with a Box–Behnken design (BBD). The TSA content (*Y*) was designated as the response variable. Three key independent variables were selected for optimization: ethanol concentration (*X*_1_), extraction time (*X*_2_), and material-to-liquid ratio (*X*_3_). The corresponding experimental factors and their levels are detailed in Table 1.

The relationship between the response and the independent variables was approximated using a second-order polynomial model, expressed as

$$Y=\lambda_{0}+\sum_{i=1}^{3}\lambda_{i}X_{i}+\sum_{i<j}\lambda_{ij}X_{i}X_{j}+\sum_{i=1}^{3}\lambda_{ii}X_{i}^{2}$$

where *Y* represents the predicted response (TSA content), λ_0_ is the constant coefficient, λ_i_ are the linear coefficients, λ_ij_ are the quadratic coefficients, λ_ii_ are the interaction coefficients, and *X*_i_ and *X*_j_ denote the coded levels of the independent variables.

### 2.7. Sensory Review of Instant Black Tea

Sensory review of instant black tea was performed following the previous report [24]. Briefly, 0.5 g of instant black tea powder was weighed into 250 mL transparent glass cups and infused with 150 mL of purified water at 85 ± 5 °C. A trained seven-member panel assessed the sensory attributes of the samples, including appearance, liquor color, aroma, taste, and solubility. Each attribute was scored on a 100-point scale. The final sensory score was calculated as the weighted average of all panelists’ ratings. Detailed evaluation criteria are provided in Table 2. The panel size was chosen based on common practice for descriptive sensory analysis of tea products; no formal sample size calculation was performed given the exploratory nature of this evaluation.

### 2.8. Statistical Analysis

All experiments were conducted in triplicate, and results were expressed as mean ± standard deviation. Data processing was carried out using Excel 2016 (Microsoft Corp., Redmond, WA, USA). One-way analysis of variance (ANOVA) was performed, and graphical visualization was generated using Origin 2022 (OriginLab Corp., Northampton, MA, USA). Response surface methodology (RSM) for experimental design and optimization was implemented via Design-Expert 12 (Stat-Ease Inc., Minneapolis, MN, USA). Statistical analyses were conducted using SPSS Statistics 27 (IBM Corp., Armonk, NY, USA), with significance defined as *p* < 0.05.

## 3. Results and Discussion

### 3.1. Optimization of Preparation Parameters

**Figure 1:**
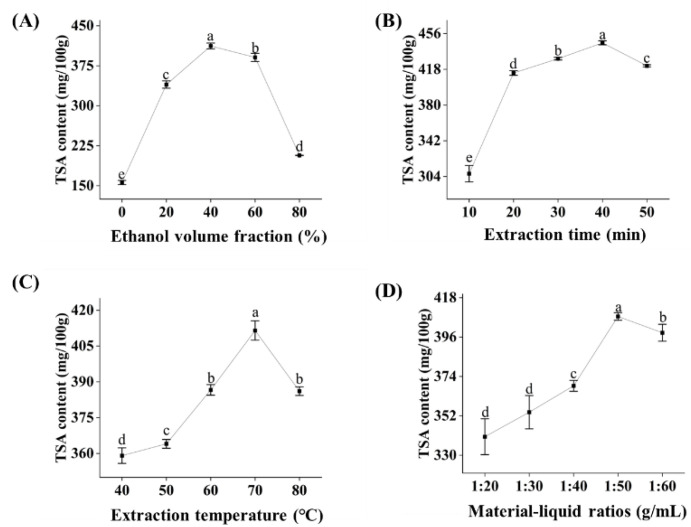
TSA content in prepared instant black tea under optimization of extraction parameters, including (**A**) ethanol volume fraction, (**B**) extraction time, (**C**) extraction temperature, and (**D**) material-liquid ratios. Letters above the points indicate the significant difference in the results.

To maximize the retention of TSA in instant black tea, key extraction parameters were systematically optimized using single-factor experiments, with TSA content serving as the primary quality indicator. As shown in Figure 1A, TSA content initially increased and then declined across the tested ethanol concentration range (0–80%, *v*/*v*). The maximum TSA content (412.02 mg/100 g) was obtained at 40% ethanol, beyond which further increases in ethanol concentration led to a progressive reduction in TSA yield, indicating that 40% aqueous ethanol provided optimal solubility for TSA extraction. The effect of extraction time on TSA content is illustrated in Figure 1B. TSA content increased rapidly within the first 20 min and reached a peak of 450 mg/100 g at 40 min, followed by a slight decline at 50 min. These results suggest that an extraction time of 40 min is sufficient for efficient TSA recovery. Temperature also significantly influenced TSA extraction (Figure 1C). Elevated temperatures enhanced TSA yield by promoting cell wall disruption and increasing solute solubility. The optimal extraction temperature was found to be 70 °C, while further heating to 80 °C resulted in reduced TSA content, likely attributable to thermal degradation. As presented in Figure 1D, TSA content increased with increasing material–liquid ratio, presumably due to a higher concentration gradient and enhanced mass transfer. The maximum TSA content was achieved at a ratio of 1:50 (g/mL), beyond which no significant improvement was observed. It is worth noting that the extraction parameters optimized in this study are within the rational range of conventional aqueous ethanol extraction [25]. Emerging technologies, such as simultaneous ultrasonic/microwave-assisted extraction (UMAE), have been reported to achieve high extraction efficiency for bioactive compounds [26]. Future studies could explore the application of such advanced technologies to further enhance the production efficiency of TSA-enriched instant tea.

Based on these single-factor results, the following parameter ranges were selected for subsequent response surface methodology (RSM) optimization: ethanol concentration (40–60%), extraction time (30–50 min), and material-to-liquid ratio (1:40–1:60 g/mL). This study focused on developing a novel ethanol-aqueous extraction method to produce TSA-enriched instant black tea, with the goal of demonstrating the feasibility and advantages over conventional water extraction. Several parameters that may influence TSA yield, including extraction temperature, particle size, pH, and number of extraction cycles, were not systematically optimized via RSM. Under the current conditions, TSA was extracted efficiently enough to clearly distinguish the novel preparation from the conventional one. Including these additional variables in the RSM design would have substantially increased experimental complexity. Future studies, particularly those aimed at industrial scale-up, should investigate the effects of these parameters and explore their optimal settings.

### 3.2. Response Surface Experimental Results

A three-level, three-factor Box–Behnken design (BBD) based on response surface methodology (RSM) was employed to optimize the extraction process for TSA-enriched instant black tea. The experimental design and corresponding TSA contents are presented in Table 3. Multiple regression analysis was performed to fit the response data, yielding the following second-order polynomial equation:

$$Y=449.70+21.35X_{1}+3.29X_{2}+5.85X_{3}-2.95X_{1}X_{2}+0.7623X_{1}X_{3}+0.7647X_{2}X_{3}-24.17X_{1}X_{1}-12.25X_{2}X_{2}-3.16X_{3}X_{3}$$

where Y represents the TSA content (mg/100 g), and $X_{1}$, $X_{2}$, and $X_{3}$ are the coded values of ethanol concentration (%, *v*/*v*), extraction time (min), and material-to-liquid ratio (g/mL), respectively.

**Figure 2:**
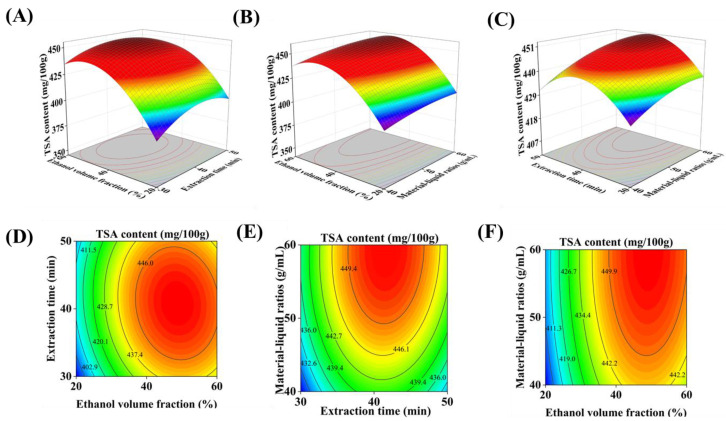
Surface and contour plots showing the interaction effects of the tested factors on TSA content in prepared instant tea. (**A**) Surface plot and (**D**) contour plot for extraction time (min) and ethanol volume fraction (%); (**B**) surface plot and (**E**) contour plot for material-liquid ratio (g/mL) and extraction time (min); (**C**) surface plot and (**F**) contour plot for material-to-liquid ratio (g/mL) and ethanol volume fraction (%).

As shown in Table 4, the regression model exhibited high statistical significance, as evidenced by an F-value of 63.10 and a *p*-value of <0.0001. The lack-of-fit was not significant (*p* > 0.05), confirming the adequacy and reliability of the model for describing the relationship between the variables and the response. Analysis of variance (ANOVA) indicated that all three linear terms ($X_{1}$, $X_{2}$, and $X_{3}$) had significant effects on TSA content (*p* < 0.05), while the quadratic terms ($X_{1}X_{1}$ and $X_{2}X_{2}$) were highly significant (*p* < 0.01). The interaction effects among the three variables were all non-significant (*p* > 0.05), as visualized in the three-dimensional response surface plots (Figure 2). The goodness-of-fit of the model was further assessed by several diagnostic parameters. The coefficient of determination (*R*^2^ = 0.9878) was well above 0.85, indicating excellent correlation between predicted and experimental values. The adjusted *R*^2^ (0.9722) demonstrated that the model explained 97.22% of the total variation in TSA content. The low coefficient of variation (CV = 0.8370) reflected high precision and reliability of the experimental data. Moreover, the difference between adjusted *R*^2^ and predicted *R*^2^ was less than 0.2, further confirming the strong predictive power of the model. These results collectively demonstrate that the established model is robust, exhibits minimal experimental error, and can be reliably used to analyze and predict TSA yield under varying extraction conditions. Based on the regression coefficients, the relative influence of the three factors on TSA content, in descending order of significance, was: ethanol concentration ($X_{1}$) > material-to-liquid ratio ($X_{3}$) > extraction time ($X_{2}$). This ranking provides practical guidance for prioritizing process adjustments in industrial production. The dominant role of ethanol concentration over other parameters has also been observed in the extraction of total polyphenols from white tea [27]. While ethanol can play a key role in industrial polyphenol extraction, the economic balance between the additional cost of solvent consumption and the benefit of elevated extract yields must be taken into account when implementing ethanol-based processes.

Using the regression model, the optimal extraction conditions for maximizing TSA content were predicted as follows: ethanol concentration of 49.01%, extraction time of 41.11 min, and material-to-liquid ratio of 1:59.94 g/mL, with a corresponding predicted TSA content of 457.60 mg/100 g. Considering operational convenience, these parameters were slightly adjusted to 49% ethanol, 41 min, and 60 g/mL. Validation experiments conducted under these adjusted conditions (*n* = 3) yielded a mean TSA content of 456.05 ± 4.45 mg/100 g, which is in close agreement with the predicted value of 457.59 mg/100 g (relative error = 0.3%). This excellent concordance confirms the high predictive accuracy and practical applicability of the developed model for optimizing TSA extraction from black tea.

### 3.3. Chemical Composition Analysis

Instant black tea powder was prepared from Jun Mei black tea using two different extraction methods: ethanol-aqueous extraction (novel instant tea preparation) and conventional hot-water extraction (traditional instant tea preparation). The chemical compositions of the raw material and the two instant tea powders are summarized in Table 5. The Jun Mei black tea contained 15.28% tea polyphenols, 0.46% TSA, 4.08% total catechins (CATE), and 0.56% theaflavins (TFs). Compared with the raw black tea, both traditional and novel instant tea powder exhibited higher proportions of tea polyphenols and individual catechin fractions. Notably, the novel instant black tea prepared by ethanol-aqueous extraction showed elevated levels of TSA, TF, TF-3-G, and TF-3′-G + TFDG. A distinct compositional difference was observed between the two processing methods. The traditional water-extracted instant tea was enriched in non-ester catechins (EGC, C, and EC), whereas the ethanol-extracted product contained significantly higher levels of ester catechins (EGCG, GCG, and ECG). This discrepancy is likely attributable to the hydrolytic effect of the traditional aqueous process, which promotes the breakdown of larger molecular structures such as EGCG and ECG. This discrepancy is likely attributable to the hydrolytic effect of the traditional aqueous process, which promotes the breakdown of larger molecular structures such as EGCG and ECG [28]. For TSA, its lower water solubility leads to greater loss during traditional water extraction compared with raw black tea [29], which is why the novel ethanol-aqueous method was developed to enhance its enrichment.

### 3.4. Sensory Evaluation

**Figure 3:**
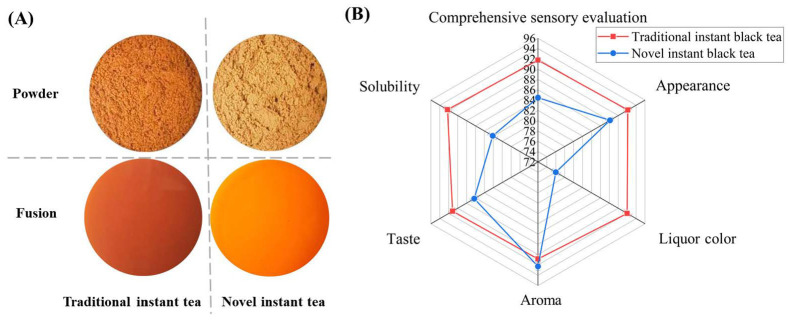
(**A**) Visual appearance of instant black tea powders and their corresponding infusions prepared by traditional (water extraction) and novel (aqueous ethanol extraction) methods; (**B**) radar chart of sensory evaluation attributes for the two instant tea powders.

As shown in Figure 3A, the traditional instant black tea exhibited a brownish-red appearance and produced an orange-red infusion. In contrast, the novel instant black tea prepared by aqueous ethanol extraction appeared dark yellow and yielded a dark brownish-red liquor. Sensory evaluation (Figure 3B) revealed that the ethanol-extracted instant tea possessed superior aroma, although its other sensory attributes differed from those of the traditional product. Specifically, the novel instant black tea displayed a darker infusion color and was characterized by a stronger, thicker, and more astringent taste profile. It is known that most volatile compounds responsible for the aroma of instant tea, including aldehydes, alcohols, ketones, and esters, are relatively nonpolar [21], which may explain the enhanced aroma intensity of the novel instant black tea, given that the less polar ethanol was used for extraction. Besides this, it should be noted that the sensory evaluation was preliminary and based on a small trained panel. Future studies should include a larger, consumer-based panel with statistical sample size calculation to validate these findings.

## 4. Conclusions

In this study, an aqueous ethanol-mediated extraction method was developed to produce instant black tea enriched with theasinensin A (TSA). Processing parameters were optimized using response surface methodology, and a multiple regression model was established to predict TSA content under varying extraction conditions. The optimal parameters for maximizing TSA yield were predicted as follows: ethanol concentration of 49.01%, extraction time of 41.11 min, and material-to-liquid ratio of 1:59.94 g/mL, corresponding to a maximum predicted TSA content of 457.60 mg/100 g. The model was experimentally validated with high accuracy, yielding a deviation of only 0.3%. The chemical composition and sensory characteristics of the novel instant black tea prepared by ethanol-aqueous extraction were systematically compared with those of traditional water-extracted instant tea. The ethanol-extracted product exhibited significantly higher levels of TSA, theaflavins (TF, TF-3-G), and TF-3′-G + TFDG, along with improved aroma quality. These findings provide a scientific basis for the development of functional instant black tea powder with enhanced bioactive profiles and offer practical guidance for process optimization in industrial applications.

## Figures and Tables

**Table 1:** Box–Behnken experimental design matrix for response surface methodology.

Coded Levels	Independent Variable		
	Ethanol Volume Fraction (%) X_1_	Extraction Time (min) X_2_	Material–Liquid Ratio (g/mL) X_3_
−1	20	30	1:40
0	40	40	1:50
+1	60	50	1:60

**Table 2:** Detailed sensory evaluation criteria for instant black tea.

Quality Factors	Sensory Score	Evaluation Criteria
Appearance (25%)	≥90	Hollow particles, wafer-like, low bulk, glossy, good dryness
	80–89	Solid grains, high capacity, glossy, good dryness
	70–79	Powdery, high capacity, non-glossy, poorly dispersible
Soup color (20%)	≥90	Orange or red color, clear and bright, no precipitate
	80–89	Orange or red color, still bright, no precipitate
	70–79	Dark and cloudy, with sediment or scum
Aroma (10%)	≥90	Strong black tea flavor and aroma
	80–89	Lighter black tea flavor and aroma
	70–79	No black tea flavor
Taste (25%)	≥90	Rich flavor, fresh and tasty
	80–89	Moderately tasty, still palatable
	70–79	Bland flavor and poor texture
Solubility (20%)	≥90	Dissolves quickly
	80–89	Completely soluble in water after stirring
	70–79	Poor solubility, scum on the surface of the water, sediment at the bottom of the glass

**Table 3:** Box–Behnken experimental design matrix with corresponding TSA content (response variable) for process optimization.

Run	Independent Variables			Responses
	X_1_ (%)	X_2_ (min)	X_3_ (mL/g)	Y (mg/100 g)
1	20	30	1:50	389.71
2	40	30	1:40	422.99
3	40	30	1:60	434.53
4	60	40	1:50	434.76
5	20	40	1:40	394.84
6	20	40	1:60	403.64
7	40	40	1:50	449.13
8	40	40	1:50	446.72
9	40	40	1:50	452.74
10	40	40	1:50	448.68
11	40	40	1:50	451.23
12	60	40	1:40	439.57
13	60	40	1:60	451.42
14	20	50	1:50	397.69
15	40	50	1:40	432.52
16	40	50	1:60	447.13
17	60	50	1:50	430.95

Note: Y, yield of theasinensin A; X_1_, X_2_ and X_3_ represent ethanol volume fraction, extraction time and material–liquid ratio, respectively.

**Table 4:** Analysis of variance (ANOVA) for the response surface quadratic regression model.

Source	SS	d.f.	MS	*F*-Value	*p*-Value	
Model	7392.80	9	821.42	63.10	<0.0001	**
X_1_	3647.45	1	3647.45	280.20	<0.0001	**
X_2_	86.38	1	86.38	6.64	0.0367	*
X_3_	273.74	1	273.74	21.03	0.0025	**
X_1_X_2_	34.79	1	34.79	2.67	0.1461	NS
X_1_X_3_	2.32	1	2.32	0.1786	0.6853	NS
X_2_X_3_	2.34	1	2.34	0.1797	0.6844	NS
X_1_X_1_	2460.25	1	2460.25	189.00	<0.0001	**
X_2_X_2_	631.70	1	631.70	48.53	0.0002	**
X_3_X_3_	41.96	1	41.96	3.22	0.1157	NS
Residual	91.12	7	13.02	-	-	-
Lack-of-fit	69.35	3	23.12	4.25	0.0981	NS
Pure error	21.77	4	5.44	-	-	-
Cor total	7483.93	16	-	-	-	-
C.V.	-	-	0.8370	-	-	-
*R* ^2^	-	-	0.9878	-	-	-
Adjusted *R*^2^	-	-	0.9722	-	-	-
Predicted *R*^2^	-	-	0.8472	-	-	-

Note: *X*_1_, *X*_2_ and *X*_3_ represent ethanol volume fraction, extraction time and material–liquid ratio, respectively; SS, d.f., MS and C.V. represent sum of squares, degrees of freedom, mean square, and coefficient of variation, respectively; ** represents *p* < 0.01; * represents *p* < 0.05. NS, not significant at *p* > 0.05.

**Table 5:** Chemical composition of Jun Mei black tea and instant tea powders prepared by hot-water and aqueous ethanol extraction. Superscript letters indicate the significant difference between the three black teas in the results.

Mass Ratio (%)	Jun Mei Black Tea	Traditional Instant Black Tea	Novel Instant Black Tea
Total polyphenols	15.28 ± 0.74 ^c^	23.45 ± 0.70 ^b^	31.09 ± 0.78 ^a^
GA	0.4 ± 0.01 ^c^	1.28 ± 0.04 ^a^	0.94 ± 0.03 ^b^
EGC	0.41 ± 0.02 ^c^	1.54 ± 0.03 ^a^	0.92 ± 0.04 ^b^
C	0.14 ± 0.00 ^c^	0.38 ± 0.01 ^a^	0.20 ± 0.01 ^b^
CAF	3.26 ± 0.05 ^b^	8.08 ± 0.13 ^a^	8.12 ± 0.15 ^a^
TSA	0.46 ± 0.01 ^b^	0.29 ± 0.01 ^c^	0.92 ± 0.01 ^a^
EC	0.32 ± 0.01 ^c^	0.98 ± 0.03 ^a^	0.77 ± 0.03 ^b^
EGCG	1.60 ± 0.07 ^c^	2.87 ± 0.05 ^b^	3.20 ± 0.03 ^a^
GCG	0.30 ± 0.01 ^c^	0.56 ± 0.02 ^b^	0.67 ± 0.04 ^a^
ECG	1.16 ± 0.04 ^c^	1.83 ± 0.03 ^b^	2.55 ± 0.01 ^a^
CG	0.15 ± 0.00 ^c^	0.35 ± 0.01 ^a^	0.32 ± 0.01 ^b^
TF	0.05 ± 0.00 ^b^	0.03 ± 0.00 ^c^	0.09 ± 0.00 ^a^
TF3 G	0.13 ± 0.00 ^b^	0.05 ± 0.00 ^c^	0.26 ± 0.01 ^a^
TF3′G + TFDG	0.38 ± 0.01 ^b^	0.09 ± 0.00 ^c^	0.78 ± 0.03 ^a^
CATE	4.08 ± 0.14 ^b^	8.52 ± 0.11 ^a^	8.63 ± 0.06 ^a^
TFs	0.56 ± 0.01 ^b^	0.17 ± 0.00 ^c^	1.11 ± 0.04 ^a^

Note: CATE represents the sum of EGC, C, EC, EGCG, GCG, ECG and CG; TFs represent the sum of TF, TF3G, TF3′G and TFDG.

## Data Availability

The original contributions presented in the study are included in the article, further inquiries can be directed to the corresponding author.

## Abbreviations

The following abbreviations are used in this manuscript:

CAF	caffeine
GA	gallic acid
GC	gallocatechin
EGC	epigallocatechin
C	catechin
EC	epicatechin
EGCG	epigallocatechin gallate
GCG	gallocatechin gallate
ECG	epicatechin gallate
CG	catechin gallate
TF	theaflavin
TF-3-G	theaflavin-3-gallate
TF-3′-G	theaflavin-3′-gallate
TFDG	theaflavin-3,3′-digallate
TSA	theasinensin A
